# Real-time integration between Microsoft HoloLens 2 and 3D Slicer with demonstration in pedicle screw placement planning

**DOI:** 10.1007/s11548-023-02977-0

**Published:** 2023-06-13

**Authors:** Alicia Pose-Díez-de-la-Lastra, Tamas Ungi, David Morton, Gabor Fichtinger, Javier Pascau

**Affiliations:** 1 Departamento de Bioingeniería, Universidad Carlos III de Madrid, 28911 Leganés, Spain; 2 Laboratory for Percutaneous Surgery, School of Computing, Queen’s University, Kingston, ON K7M2N8 Canada

**Keywords:** Augmented reality, Microsoft HoloLens 2, OpenIGTLink, 3D Slicer, Pedicle screw, Surgical planning

## Abstract

**Purpose:**

Up to date, there has been a lack of software infrastructure to connect 3D Slicer to any augmented reality (AR) device. This work describes a novel connection approach using Microsoft HoloLens 2 and OpenIGTLink, with a demonstration in pedicle screw placement planning.

**Methods:**

We developed an AR application in Unity that is wirelessly rendered onto Microsoft HoloLens 2 using Holographic Remoting. Simultaneously, Unity connects to 3D Slicer using the OpenIGTLink communication protocol. Geometrical transform and image messages are transferred between both platforms in real time. Through the AR glasses, a user visualizes a patient’s computed tomography overlaid onto virtual 3D models showing anatomical structures. We technically evaluated the system by measuring message transference latency between the platforms. Its functionality was assessed in pedicle screw placement planning. Six volunteers planned pedicle screws' position and orientation with the AR system and on a 2D desktop planner. We compared the placement accuracy of each screw with both methods. Finally, we administered a questionnaire to all participants to assess their experience with the AR system.

**Results:**

The latency in message exchange is sufficiently low to enable real-time communication between the platforms. The AR method was non-inferior to the 2D desktop planner, with a mean error of 2.1 ± 1.4 mm. Moreover, 98% of the screw placements performed with the AR system were successful, according to the Gertzbein–Robbins scale. The average questionnaire outcomes were 4.5/5.

**Conclusions:**

Real-time communication between Microsoft HoloLens 2 and 3D Slicer is feasible and supports accurate planning for pedicle screw placement.

**Supplementary Information:**

The online version contains supplementary material available at 10.1007/s11548-023-02977-0.

## Introduction

Effective preoperative planning is crucial to the success of surgical procedures. Various simulation and visualization techniques have been developed in recent years to help surgeons better prepare for interventions. While medical images provide information about patient anatomy, their 2D nature limits depth perception [1]. To address this shortcoming, 3D displays, and related technologies have gained popularity in helping to understand patient conditions better [2].

3D Slicer is widely used for medical image visualization and analysis [3]. This free, open-source software offers a range of capabilities for surgical planning, intraoperative guidance, and clinical research [4, 5]. Its compatibility with OpenIGTLink, a standardized network communication protocol for image-guided treatments, is a key feature that has allowed connecting commercial navigation systems or robots to guide clinical procedures [6]. To facilitate this interconnection, Lasso et al. [7] developed PLUS (public software library for ultrasound imaging research) that acquires data from different tracking systems and sends it to any client, including 3D Slicer, using OpenIGTLink.

Alternative 3D techniques, such as virtual reality (VR) and augmented reality (AR), have been successfully adopted in various clinical applications [8]. When used for long periods, VR headsets may induce headaches, dizziness, or blurred vision [9]. In contrast, the spatial awareness provided by AR headsets minimizes these adverse effects. As a result, it seems reasonable to prioritize this technology for long-lasting tasks such as surgical planning or training. AR has been successfully adopted alone and in combination with other techniques (such as 3D printing) in multiple scenarios, including surgeries [10]. It can be deployed on inexpensive smartphones or tablets, but they present a limited field of view and do not allow interaction with virtual information. When AR is deployed on head-mounted displays (HDM-AR) ergonomics are improved thanks to voice and gesture recognition. Microsoft HoloLens 2 is currently the most widely used HMD-AR device in clinical settings [11]. Microsoft HoloLens visualization has been commonly referred to as mixed reality (MR). However, in this paper, we will refer to any technology that combines virtual and real worlds in visualization as AR [12].

One of the primary drawbacks of HDM-AR is their limited processing power, which restricts the complexity of applications that can be run on these devices [13]. A solution would be to process medical images in 3D Slicer and transfer the results to the HMD-AR device. However, there is no software infrastructure for transferring information from 3D Slicer to AR devices. The aim of the study is twofold: first, establish a connection between 3D Slicer and Microsoft HoloLens 2 using the OpenIGTLink communication protocol. This integration combines the strengths of both tools to improve visualization and interaction with medical images. Specifically, we developed a system on which the AR glasses display computed tomography (CT) slices received from 3D Slicer in real-time. Our second goal was to demonstrate its functionality in a medical context. As an example, we contemplate pedicle screw placement planning, a critical surgical procedure that treats a range of conditions by fixing the spine with pedicle screws.

Our approach demonstrates the potential of AR to improve surgical planning by providing an immersive and interactive visualization of medical image data. Our project is based on the work presented in [14]. They displayed ultrasound (US) images in Microsoft HoloLens 2 utilizing the PLUS toolkit as the communicator between the AR glasses and a US probe. In our case, we aimed to generalize the type of images shown in the AR glasses using 3D Slicer as the image processor and emitter. All source code and material used in this study are publicly available at https://github.com/BSEL-UC3M/HoloLens2and3DSlicer-PedicleScrewPlacementPlanning.git.

## Materials and methods

The current section is organized into subsections that correspond to the two main objectives of this work: “Technical developments” and “Use case: pedicle screw placement planning”. Within the first subsection, “System overview” describes the AR system developed for this project. “3D Slicer modules” introduces the modules implemented in 3D Slicer. In “Microsoft HoloLens 2 application”, we discuss the AR application we developed in Unity for Microsoft HoloLens 2. On the other hand, the subsection “Use case: pedicle screw placement planning” is subdivided in “Background”, which provides some context regarding the clinical use case we employed to analyze the system’s functionality. The subsection also contains details about the patients under study, experimental setup, and functional evaluation in “Dataset”, “Experimental setup”, and “Methods of technical and functional evaluation”, respectively.

### Technical developments

#### System overview

**Fig. 1:**
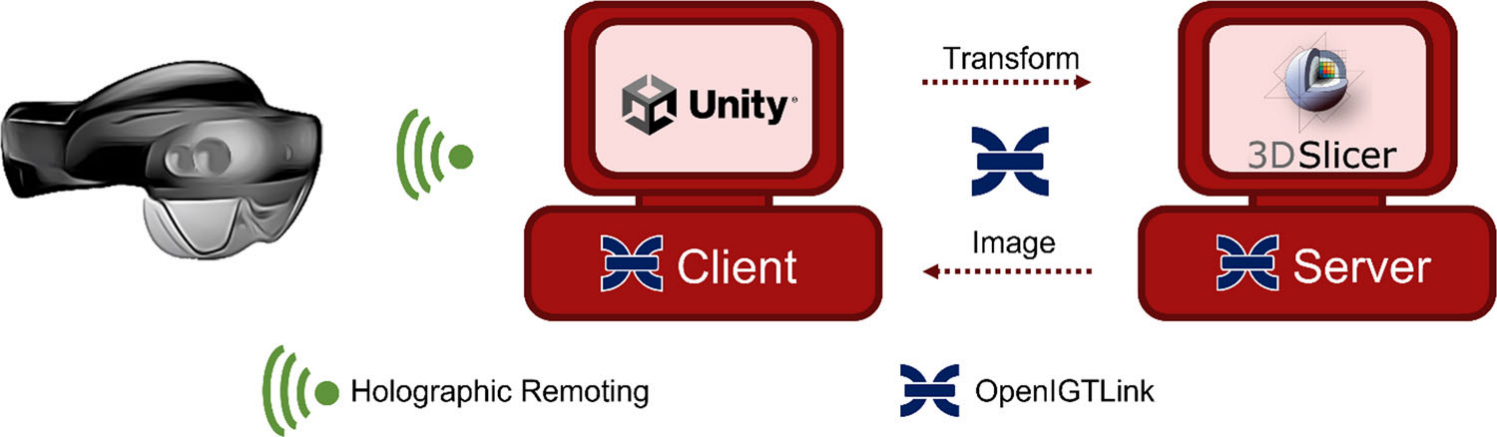
Schematic overview of the AR system

Our AR system includes three main components: A Microsoft HoloLens 2 device, the Unity platform, and the 3D Slicer software (Figure 1). The latter two can run on the same computer or separately. Unity is the core of the system and contains the AR application. When executed, it streams the content into Microsoft HoloLens 2 via Holographic Remoting with the device’s IP address [15]. The test application we developed for pedicle screw placement planning starts with a virtual 3D model of a patient’s spine, a control panel with buttons to manipulate the virtual models, and a square plane with a handler (CT image plane). Pedicle screws of varying dimensions can be easily added to the scene using voice commands. All models can be freely moved in the 3D world with intuitive hand gestures.

Unity and 3D Slicer are connected using OpenIGTLink communication protocol. We created an OpenIGTLink server in 3D Slicer using the OpenIGTLinkIF module. Unity has no library supporting this protocol, so we developed a custom-made client-side extending the code provided at https://github.com/franklinwk/OpenIGTLink-Unity. This code creates a TCP/IP socket that finds the server through a known hostname and port. Our pedicle screw planning application uses this communication bridge to transfer geometrical transforms and images. Whenever the user moves any model in the 3D world from Microsoft HoloLens 2, the updated pose information is received in Unity. The geometric transforms corresponding to the spine, CT plane, and pedicle screws are automatically sent to 3D Slicer. In Microsoft HoloLens 2, the origin of coordinates is established at the center of the user’s head when the Holographic Remoting app is initialized. All the geometrical transform information sent is in that local reference frame, considering the relative displacements of the models.

Once 3D Slicer receives this information, it loads analogous 3D models of the spine and each screw and applies the corresponding transforms on its local frame (irrelevant to our task). The information required to load the models of interest from the local storage of the PC is included in the message’s metadata. 3D Slicer reslices the patient’s CT (preloaded) according to the CT image plane pose with respect to the spine model. The resulting 2D image is sent back to Unity and Microsoft HoloLens 2 through the same communication channel.

**Fig. 2:**
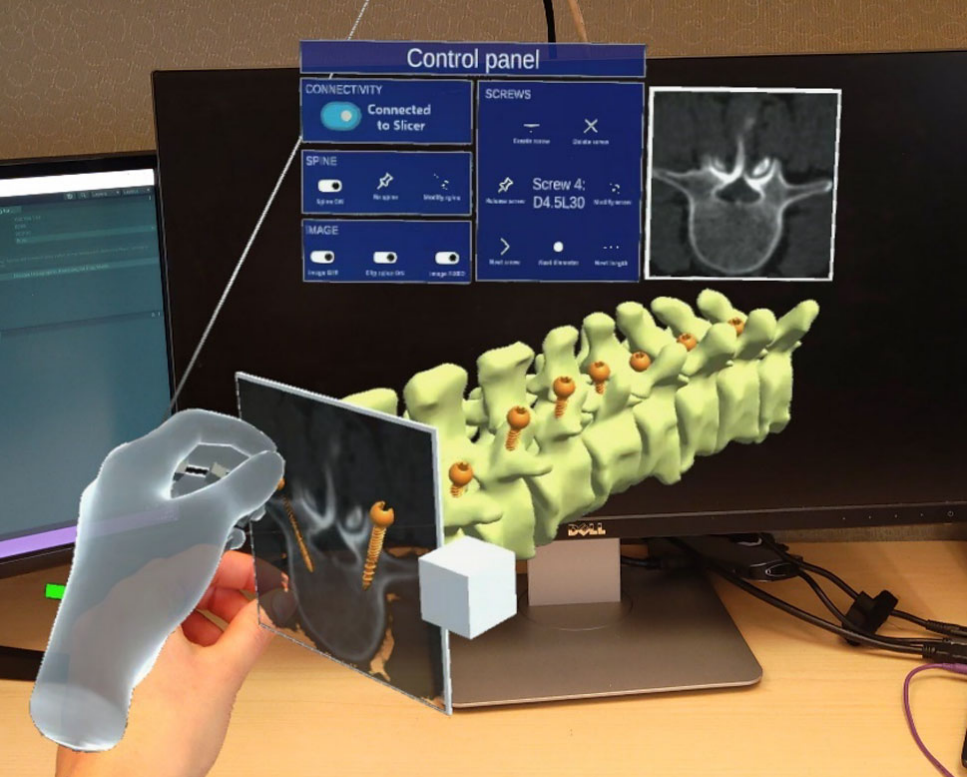
AR application for pedicle screw placement planning

These features allow users to seamlessly manipulate the CT plane along the patient’s spine to view the corresponding resliced CT image in real-time. The main advantage of planning with no registration with real elements is that 3D models can be scaled and rotated freely to find the best perspective for inserting each screw. The final appearance of the application is shown in Fig. 2.

#### 3D Slicer modules

We developed two personalized modules on 3D Slicer version 5.0.3. The first one complements the AR system. It processes any CT volume to create a resliced 2D image of 100 × 100 uint8 pixels. The 2D slice can be freely moved within the CT volume and the module interpolates the 2D image corresponding to the intersection of the image plane and the 3D volume. This module also creates an OpenIGTLink server and sends the 2D slice to any potential client (Unity, in our case).

**Fig. 3:**
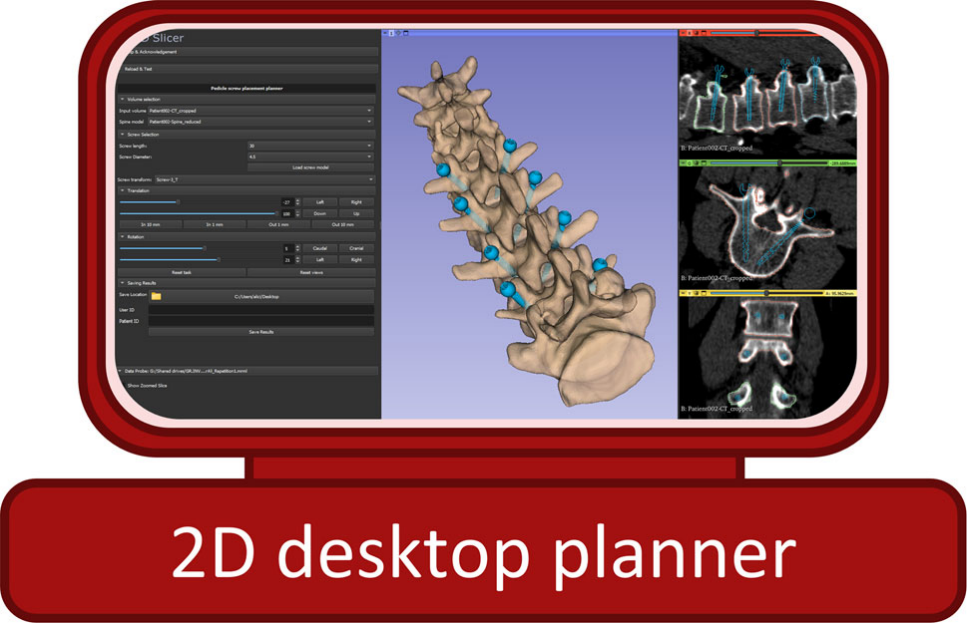
3D Slicer module for pedicle screw placement 2D desktop planning

The second 3D Slicer module simulates a traditional desktop planner for pedicle screw placement (Fig. 3). It first loads a patient’s CT volume and the corresponding 3D model of their spine. The user can then create new screws specifying their thickness and length. The module incorporates several sliders and buttons to translate and rotate the pedicles in the 3D view. It also shows the sagittal, axial, and coronal planes of the volume with the silhouettes of each screw to facilitate the planning process.

#### Microsoft HoloLens 2 application

**Fig. 4:**
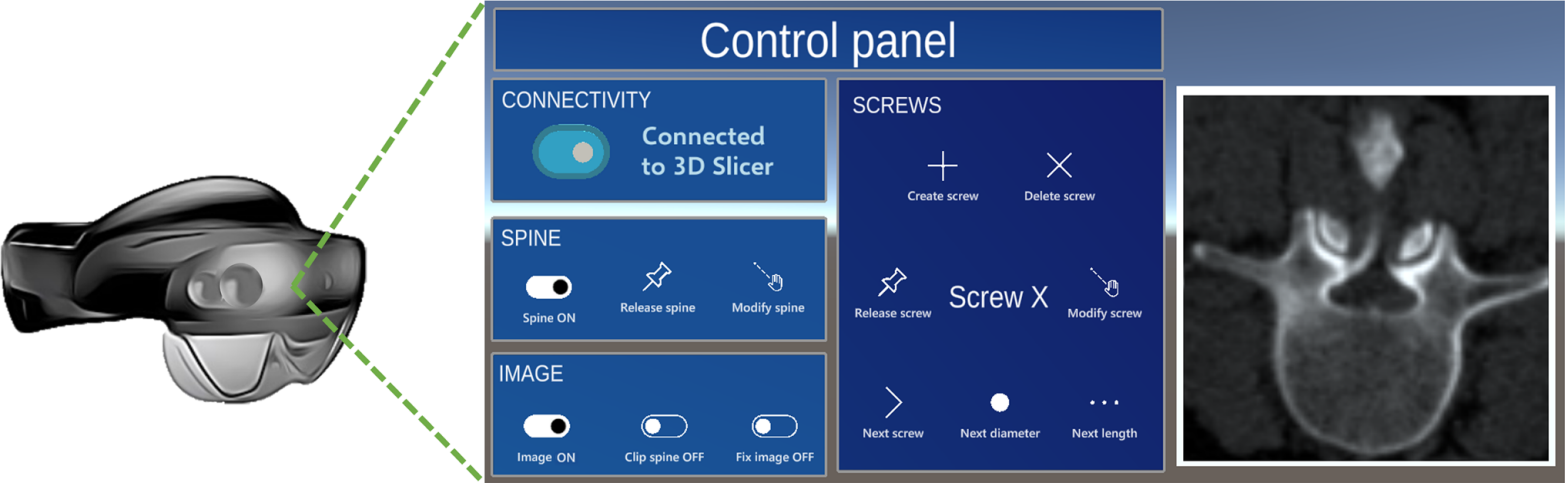
User interface of the AR application for pedicle screw placement planning

We developed the AR application in Unity 2021.3.9f1 using C# programming language and Unity Mixed Reality Toolkit (MRTK) library (https://github.com/Microsoft/MixedRealityToolkit-Unity). Apart from the virtual 3D models of the spine, the CT image plane, and the screws, the application includes a control panel (Fig. 4). The buttons turn on/off the visibility of the associated virtual models and enable/disable their manipulation in the 3D world. We included a tool to clip the spine with the resliced 2D image (recall Fig. 2). This allows seeing the patient’s CT slice in situ. The virtual screws can have different dimensions. Buttons *Next screw*, *Next diameter*, and *Next length* iterate over all the screws in the scene and all possible sizes to modify their parameters as desired.

3D models change color to indicate whether they are editable or not. Voice commands can be used to activate the buttons, which are labeled with the corresponding command text. This allows for smooth interaction with the virtual models, as the user can speak the desired action (e.g., “create screw” or “modify spine”).

### Use case: pedicle screw placement planning

#### Background

Most spinal deformities caused by scoliosis, fractures, tumors, and many degenerative diseases, are treated with pedicle screws. This procedure involves fixing the spine with screws that provide strength and scaffolding for a bony fusion [16]. Pedicle screws are posteriorly inserted through the pedicles in the vertebral bodies. Traditionally, this procedure has been performed with a free-hand technique using anatomical landmarks to guide the appropriate entry point, trajectory, and depth. However, this is a challenging procedure due to the proximity of sensitive anatomical structures. Improper screw placement can result in vascular and nerve injuries, as well as compromised screw retention and short-term implant failure [17].

Some criteria have been established to ensure proper placement [18]. First, the entry point should follow the spinal curvature. Second, the trajectory should coincide with the pedicle when a straightforward insertion technique is followed, although there are other alternative methods such as the anatomic trajectory, useful in salvage situations [19]. Still, there is no universal approach, and the final positioning highly depends on the surgeon’s preferences. Computer algorithms, fluoroscopy guidance, and robot-assisted techniques are some solutions proposed to objectify these procedures [20, 21]. Despite their benefits, these techniques are subject to line-of-sight interruption and attention shift. Alternatively, AR can enhance spinal instrumentation efficiency, safety, and accuracy [22]. Given the need to develop effective pedicle planning methods, we propose a novel AR system to facilitate and optimize these procedures. Our objective is to make them as comfortable, tolerable, and accurate as possible.

#### Dataset

We retrieved the CT scans of two healthy human spines from the fully open-access VerSe (Large Scale Vertebrae Segmentation Challenge) dataset (https://github.com/anjany/verse) [23]. The dataset also contains the segmentations for each vertebra. We combined these vertebral masks in 3D Slicer to create 3D models of each spine. These CT scans were also used to create resliced CT images to be streamed to Microsoft HoloLens 2. Patient001 and Patient002 in this study correspond to patients sub-verse768 and sub-verse760 in the VerSe20test dataset.

We focused on vertebrae from L4 to L1 because lumbar segment pedicles are the largest in the spine. This results in a higher success rate [24, 25]. Considering both patients, the average width of their pedicles is 12.3 ± 0.8 mm. The mean pedicle height is 17.1 ± 1.0 mm. We defined the pedicle’s width and height as its narrowest dimension in the transverse and sagittal planes. These values were used to effectively assess placement accuracy, considering the mean size of the pedicles and the deviation of the screws.

#### Experimental setup

Six users with diverse backgrounds volunteered to participate in our data acquisition process. This group included three undergraduate students actively engaged in surgical navigation research, one first-year medical student, and two highly experienced clinicians with over ten years of expertise in orthopedic surgery. The selection of such a varied group was intended to test the impact of experience on our technology while also gathering feedback from users with a range of different perspectives.

Each participant virtually instrumented two spines with eight screws (two in each vertebra from L4 to L1), yielding a total of 96 virtual trajectory plans. One plan was completed using the AR system (AR method), and the other using the 2D desktop planner (desktop method). The order of the methods and the spines instrumented with each technique were randomized for each user. With this, we tried to minimize the influence of any learning curve or patient-specific factors in the results. This experiment is designed based on the work published by Ungi et. al. in 2013 [24].

To enhance the realism of the planning process, the screws utilized in this study were available in three widths (Ø 4.5 mm, Ø 5 mm, and Ø 6 mm) and seven lengths (30 mm, 35 mm, 40 mm, 45 mm, 50 mm, 55 mm, and 60 mm). Nevertheless, the selection of screw dimensions was not evaluated in this study, as this criterion highly depends on the surgeon's preferences [20].

#### Methods of technical and functional evaluation

To ensure effective communication across platforms, it is crucial that the exchange of information is seamless and allows for real-time perception. In line with the approach taken by [14], we measured latency by counting the number of frames it took from a change in one of the platforms to be reflected in the other one in a slow-motion video. The video was captured at a rate of 120 Hz through the glasses, where both the holographic information and the 3D Slicer screen containing the analogous models were always visible. We focused on two distinct time ranges. Firstly, we counted the number of frames from the movement of the CT plane in the glasses to the display of the corresponding CT image in the headset (*complete transmission*). This process entailed receiving the new transform in 3D Slicer, reslicing the CT volume, and sending the image back to HL2. Second, we paid closer attention to the latency between the reslicing of the CT volume in 3D Slicer and its display in the glasses (*image reception*). This second analysis, which aligns with [14], allowed us to compare our system with the existing research in the field. This experiment was repeated 30 times for each time range.

Regarding functional evaluation, we analyzed our system under three criteria. First, we compared the overall pedicle screw placement accuracy obtained with the AR method to the accuracy achieved with the 2D desktop planner. We measured accuracy by calculating the distance between the longitudinal axis of each screw and the centerline of the pedicles. To define the centerlines of the pedicles, we identified in the CT scans the coronal cross section of the pedicles where they had the minimum diameter. This is the most critical point to avoid breaches in the cortical bone. We extracted the center point of these cross sections by drawing a line in the largest transverse and longitudinal dimensions of the pedicle cross section. We considered the intersection of these lines to be the optimal point for the screw centerline.

Our second assessment method used the patient’s CT scans to grade each screw with the Gertzbein-Robbins classification [21]. Planning was considered successful if graded A or B: No breach of the cortical layer of the pedicle and deviation of the pedicular trajectory below 2 mm. Conversely, screw placement was deemed unsuccessful if the cortical layer of the pedicle was breached in any direction or the pedicle deviation was greater than 2 mm. This corresponds to grades from C to E.

The third evaluation method involved administering an experience questionnaire to all study participants. It asked participants to rate the AR application from 1 (terrible) to 5 (perfect) in different aspects. Those include comfort and fatigue after extended use, interpretability, virtual models’ quality and interaction, and the future of AR in this context. Additionally, it had questions about the specific tools and features of the app, such as CT slice visualization and voice commands. The complete questionnaire is included in *Online SupplementaryMaterial_Questionnaire*. To determine statistically significant differences among patients or methods, we conducted a Mann–Whitney U Test. Additionally, we used Kruskal–Wallis tests to assess whether diverse pedicles and participants produced significantly distinct outcomes. Finally, we conducted a non-inferiority analysis of all the data to compare the results obtained with the AR and desktop methods. The non-inferiority margin was established at $\updelta = -10\mathrm{\%}$ [26].

## Results

A demo video showing the functioning of the application is provided in *Online SupplementaryMaterial_Video*.^1^ All virtual structures are accurately aligned in the reference frame of both 3D Slicer and the glasses. In consequence, the CT reslice seamlessly matches the corresponding section of the spine, ensuring the delivery of precise and objective information.

**Fig. 5:**
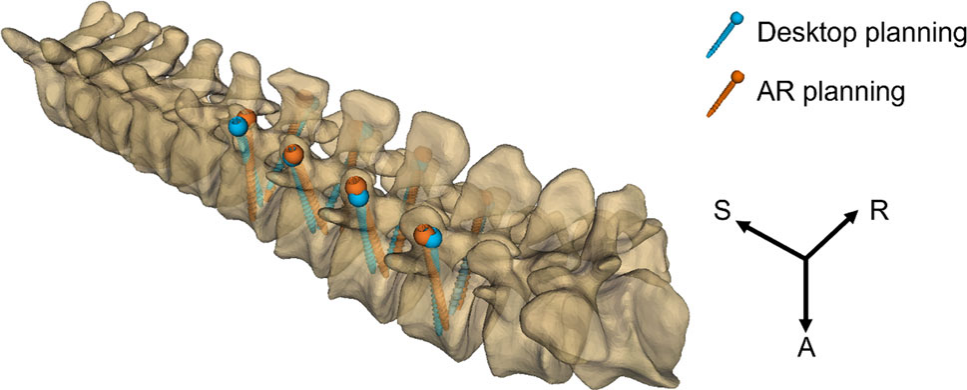
Example of 3D pedicle screw placement planning on Patient001 with the desktop (blue) and the AR methods (orange)

Five of the six volunteers had prior experience with AR in head-mounted displays and did not report any fatigue with the glasses during the entire experiment. The one who had never used AR before needed more time to adjust to the technology. Nevertheless, he performed the experiments seamlessly after 10 min of training. Figure 5 shows an example of the planning results on Patient001 using desktop and AR methods.

### Latency analysis

The *image reception* timeframe, which includes the number of frames from the creation of the CT volume reslice in 3D Slicer to the display of the new image in HL2, was completed in 2 or fewer frames in 80% of the 30 analyzed events. On the other hand, the *complete transmission*, which incorporates the selection of the plane in the glasses, concluded in 6 or fewer frames also in 80% of the cases. This translates to a maximum of 50 ms for the complete exchange of information between the platforms, dedicating a maximum of 17 ms to *image reception*.

### Pedicle screw placement planning accuracy

The results obtained with AR and desktop methods are depicted in Table 1. In all the statistical analyses performed, the p-value was greater than 0.05. This means that the patient instrumented, the user performing the experiment and the evaluated pedicle did not influence the outcomes.

**Table 1:** Pedicle screw placement error for each method

Method	Mean ± standard deviation (mm)	Median/IQR (mm)
AR	2.1 ± 1.4	1.6/1.6
Desktop	1.3 ± 0.8	1.2/0.9

In addition, we graded each screw placement following the Gertzbein-–Robbins classification (Table 2). Considering grades A–B successful and C–E unsuccessful, the overall success with the AR method is 98%. With the desktop planner, it is 100% [28]. The lower 1-sided 95% confidence limit for the AR method in the non-inferiority analysis is 0.015. This means that the AR method is non-inferior to the desktop planner. A Mann–Whitney U Test showed no statistically significant differences between the two methods using the Gertzbein–Robbins classification.

**Table 2:** Gertzbein–Robbins classification of pedicle screws for each method

Method	Screws graded A	Screws graded B	Screws graded C	Screws graded D	Screws graded E	% Successful placements
AR	35	12	0	0	1	98
Desktop	39	9	0	0	0	100

**Fig. 6:**
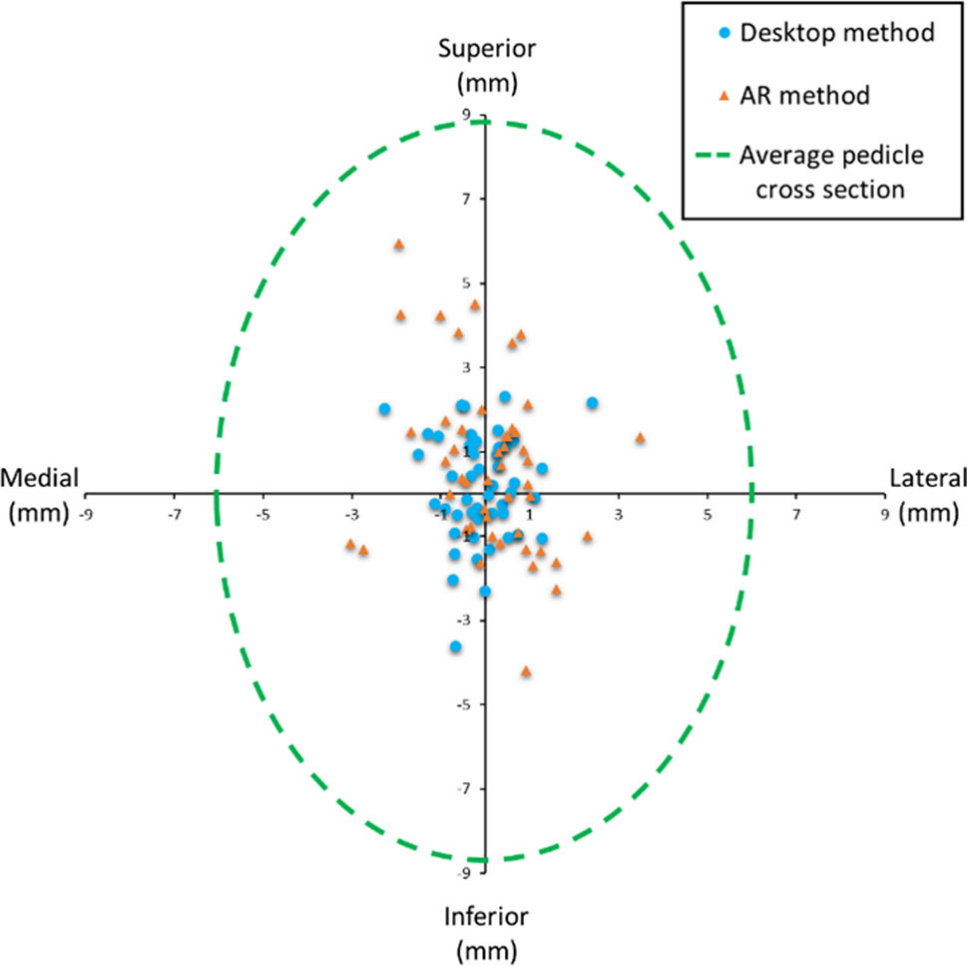
Screw placement accuracy error in the mediolateral and inferosuperior anatomical plane with AR and Desktop methods. The green dashed circle represents the average pedicle cross section in this work

Figure 6 shows a scatterplot of the placement accuracy in the mediolateral and inferosuperior anatomical planes. The green dashed line represents the average cross section of the pedicles. All points are confined within that line, meaning pedicle trajectory planning was accurate for our purposes. There is no remarkable tendency to any extreme in the mediolateral plane. In turn, there was a clear preference for inserting the screws superiorly. This is just considered a common preference since there is no consensus on the best planning approach as long as the screw does not breach the pedicle walls [20]. 81% of the screw trajectories planned with the AR method and 97% of the ones planned with the desktop method were placed within 3 mm from the center of the pedicle.

### User-experience questionnaire

**Fig. 7:**
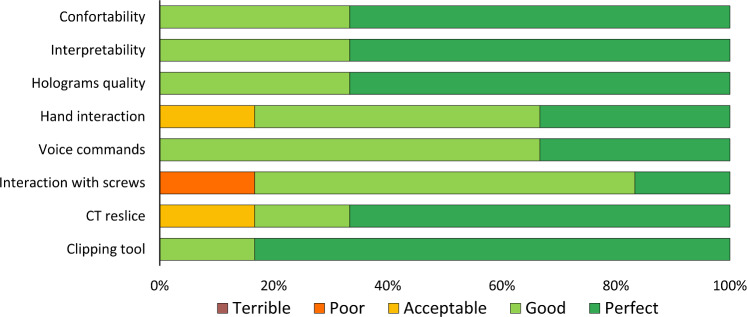
Questionnaire results

Figure 7 illustrates volunteers’ impressions of the AR method. On average, the AR application was rated at 4.5 out of 5, indicating that it was perceived as *good—perfect* for pedicle screw placement planning. All participants reported that Microsoft HoloLens 2 glasses are comfortable to wear during the planning process. They also agreed that the application was easy to understand, and the virtual models’ quality was sufficient to complete the task. The most highly regarded feature was the clipping tool that used the CT plane to trim the spine. They all reported that physically moving the plane along the spine and seeing the corresponding CT slice was very useful in improving their perception of the problem. There were no ratings of “terrible” for any of the questions on the survey.

## Discussion

One limitation of 3D Slicer-based solutions is the lack of software infrastructure for AR visualization. In this work, we developed a solution that communicates Microsoft HoloLens 2 and 3D Slicer through an OpenIGTLink client in Unity. This enables the use of the widely popular image analysis software with the famous AR device. Our objective was to exploit the 3D capabilities of the AR glasses in conjunction with image processing tools available in 3D Slicer. Our system exchanges geometrical transform and image messages between the platforms with no perceptible delay. Latency analysis revealed a close-to-real-time transference of images, with a delay of only 17 ms. This value is lower than the 80 ms reported in [27] and comparable to the 16 ms from [14]. The average time required for the complete exchange of messages was 50 ms. This process encompasses the definition of a new geometrical transform for the CT plane in the glasses to the reception of the corresponding CT reslice from 3D Slicer.

To evaluate our system’s performance in a clinical context, we tailored it for pedicle screw placement planning. Thanks to the Unity—3D Slicer communication, the application displays a virtual 2D image resliced from the CT volume in the plane selected by the user with Microsoft HoloLens 2. This allows for a more accurate visualization of the ideal screw trajectory from multiple perspectives. There is no registration of virtual models in the real world. Consequently, the models can be scaled for an enhanced understanding of the anatomy, improving confidence and spatial perception. The lack of a physical model to align with also permits clipping the spine with the selected 2D plane, enhancing the pedicle visibility from any perspective.

In parallel, we developed a more traditional desktop planner in 3D Slicer to compare with the AR application. Our results demonstrate that utilizing our AR method as a tool for pedicle screw placement planning is not only technically feasible but also accurate, with a mean error of 2.1 ± 1.4 mm. Furthermore, our system consistently yields comparable outcomes, irrespective of the user’s familiarity with AR technology or level of clinical expertise. Statistical analyses demonstrated that the accuracy achieved with our AR method was non-inferior to the one obtained with the desktop method (mean error of 1.3 ± 0.8 mm), although the outcomes of the second method were systematically better. In both cases, the results are comparable to the 1.28 ± 1.37 mm reported in [24]. In that study, the researchers used ultrasound snapshots to identify vertebral landmarks that guided the screw placement. These images, however, were displayed on an external monitor. Showing the CT slices overlaid to the spine 3D model is one of the most valuable contributions of our system.

Participants’ perspectives were also very positive on the remaining aspects of the AR system. Yet, they pondered that the best technique should combine both methods. Specifically, they would employ the AR glasses for an initial rough placement of the screws in 3D, to then fine-tune the final position with desktop controllers. Since our system sends all positions in real time from Microsoft HoloLens 2 to 3D Slicer, it is currently possible to use it following this mixed approach. Once the virtual plan has been defined, it could also be displayed alongside the patient during surgery, serving as a reference during the procedure.

One of the main limitations of the pedicle screw placement implementation is the low number of subjects validating the system. Even though the results obtained are promising, further examinations with more users could determine if previous AR experience is required to plan correctly. In addition, this proof-of-concept research did not fully explore the placement of screws in cervical or thoracic vertebrae. Future studies could expand upon this work by including planning for the complete spine in a larger group of patients. Finally, our work lacks the placement of actual screws. The reason is that we mainly focused on testing the feasibility of our system in a clinical scenario, as well as comparing its convenience to traditional planning platforms. Nevertheless, the full impact of planning errors could be better assessed in a future study with actual screw placements in phantom models, animals, or human subjects, similar to [22]. In that work, the authors present an AR system to guide pedicle screw placement with Microsoft HoloLens. They preload the patient’s CT on the AR device with the *Novarad OpenSight* application and overlay it to a silicone phantom. However, this requires uploading one heavy CT volume into the AR glasses for each patient. Moreover, it is specific to Microsoft HoloLens and cannot be easily transferred to other AR devices.

In turn, our system is designed to send and receive all necessary information in real time from the computer. This approach reduces the computational load on the AR device. Besides, any device compatible with Holographic Remoting or a similar toolkit integrated with Unity would work with our approach, enabling the transfer of the technology to other HMDs. This allows for a quick adjustment to technological advances in the future. Furthermore, since everything is developed for the computer, new patient information can be rapidly loaded anytime. It would only be necessary to import the desired 3D models into Unity and the corresponding CT volume into 3D Slicer. There is no need to build any application into the AR glasses.

After evaluating both participant feedback and accuracy outcomes, we can conclude that our AR system may not be the optimal solution for enhancing pedicle screw placement procedures, as existing technologies are equally or more accurate. However, it was not originally developed with the specific goal of enhancing pedicle screw placement procedures. Instead, we designed the experiments to test the applicability of our framework in the clinical setting. Having demonstrated its ease of use and portability to a medical context, we believe that the true value of our system lies in its potential to benefit a broad range of medical applications. By utilizing AR as the 3D visualization technology, we can expand the possibilities beyond what is achievable with other 3D techniques such as VR. This unlocks new opportunities to enhance medical procedures, including surgical interventions. As such, we are eager to explore additional use cases with our AR solution and discover new ways to enhance medical care beyond our initial focus on pedicle screw placement planning.

An example of our system's potential for future expansion is its integration with other devices compatible with OpenIGTLink, such as tracking systems. By utilizing the PLUS toolkit and this communication protocol, it is already possible to easily connect most external tools to 3D Slicer. The same channel could be employed to send information between all platforms, allowing for real-time display of tracking information directly in Microsoft HoloLens 2. This workflow could also facilitate the connection of multiple HMDs to 3D Slicer from various Unity projects. Information could be exchanged between the headsets to observe how a user manipulates a virtual model from another user’s glasses. To finish, some of the previous works did not share the data and code used for their evaluation [18, 20, 21]. We expect that providing all code and materials, following Open Science guidelines, will enable other researchers to build up on top of our solution.

## Conclusions

In this work, we used OpenIGTLink to communicate Microsoft HoloLens 2 and 3D Slicer with real-time perception. The function of the system was demonstrated for pedicle screw placement planning. The final application allows to move a virtual plane along a virtual 3D model of a patient’s spine and visualize the corresponding resliced 2D image from the CT scan. This dynamic visualization, coupled with the ability to manipulate the virtual models, enables users to easily develop surgical plans. The accuracy tests, statistical analyses, and user feedback results are very promising and comparable to the state-of-the-art in pedicle screw planning, although our main contribution is the easy transferability of our system to other clinical applications in the future. Combining AR and 3D Slicer can transform how we approach medical procedures. We hope this work is a foundation for future studies integrating AR visualization and powerful image analysis software in the medical field.

### Supplementary Information

Below is the link to the electronic supplementary material.

Supplementary file1 (DOCX 32 kb)

Supplementary file2 (MP4 323574 kb)

## Footnotes

1 The video is also available at: https://youtu.be/35WiSceP94Q
