# Supplementary material for: Real-time integration between Microsoft HoloLens 2 and 3D Slicer with demonstration in pedicle screw placement planning
Source: Int J Comput Assist Radiol Surg. 2023 Jun 13;18(11):2023–32. doi: 10.1007/s11548-023-02977-0 (PMC10589185; doi:10.1007/s11548-023-02977-0)
Supplement: Supplementary file 1 — Supplementary file1 (DOCX 32 kb) [file 11548_2023_2977_MOESM1_ESM.docx]

Supplementary material Questionnaire

Survey to evaluate the AR system that couples HoloLens 2 and 3D Slicer using OpenIGTLink

The aim of this survey is to evaluate the effectiveness of using **augmented reality (AR) in pedicle screw placement planning**. To achieve this, we have developed an AR application for Microsoft HoloLens 2.

The application allows users to view a 3D model of a **human spine** (including thoracic and lumbar vertebrae) in front of their eyes. Users can manipulate the position, rotation, and scale of the spine using intuitive hand gestures that improve their 3D perception of the model.

In addition to viewing the spine, the application allows users to create and manipulate **pedicle screws**. Users can interact with the screws in the same way they do with the spine, placing them in suitable positions with respect to the bone. They can also adjust the thickness and length of each screw and delete them as needed.

**Microsoft HoloLens 2** can be connected to imaging software (3D slicer) to retrieve the CT scan of the corresponding patient. A **CT slide** is displayed **as a hologram** overlaid on the spine model, providing additional guidance in planning the placement of the screws.

All participants placed 8 screws in a spine using the AR system and 8 screws in another spine using a desktop-planar software. Our goal is to determine whether augmented reality can improve accuracy and/or confidence of users during pedicle screw positioning planning compared to traditional approaches.

Number of questions: 12

Estimated time to complete the survey: **4 minutes**

| Name |  |
| --- | --- |
| Date |  |
| Occupation |  |
| Have you ever used augmented reality (AR) before? | No  Yes, on smartphones  Yes, on head mounted displays (AR glasses) |

| **Question** | | **Rating** | | | | | | |
| --- | --- | --- | --- | --- | --- | --- | --- | --- |
| MICROSOFT HOLOLENS 2 – TECHNICAL ASPECTS | | | | | | | | |
| 1. Evaluate the **comfortability** (in general) of the device. | | Terrible | - | | -/+ | | + | Perfect |
| 1. Evaluate the HoloLens’ fit, comfort and hold **over time** | | Terrible | - | | -/+ | | + | Perfect |
| 1. Have you experienced **fatigue** during the experiment due to the holographic view? If so, how much time were you wearing the glasses? | | I didn’t experience fatigue | 0-10 min | | 10-30 min | | 30-60 min | During the whole experiment |
| MICROSOFT HOLOLENS 2 – AR APP | | | | | | | | |
| 1. Information displayed on HoloLens was easy to **interpret** | | Strongly disagree | - | | -/+ | | + | Strongly agree |
| 1. I consider the **hologram's quality** good enough for this task | | Strongly disagree | - | | -/+ | | + | Strongly agree |
| 1. **Hand interaction** with holograms is intuitive / realistic | | Strongly disagree | - | | -/+ | | + | Strongly agree |
| 1. I find the **voice commands** very helpful for this task | | Strongly disagree | - | | -/+ | | + | Strongly agree |
| 1. Rate the overall interaction with the **screw** models | | Terrible | - | | -/+ | | + | Perfect |
| 1. I find the visualization of the holographic **CT slide** useful for surgical planning | | Strongly disagree | - | | -/+ | | + | Strongly agree |
| 1. Evaluate the tool that **clips the spine** model with the CT image | | Completely useless | - | | -/+ | | + | Very useful |
| GENERIC QUESTIONS | | | | | | | | |
| 1. Do you consider this technology is **promising** for the future of pedicle screw placement planning? | | Strongly disagree | - | -/+ | | + | | Strongly agree |
| 1. Which **method** would you rather use in your next planning? | | AR based method only  Planar desktop (traditional) method only  AR + desktop (initialize planning with HoloLens and refine with desktop) | | | | | | |
| Observations |  | | | | | | | |
